# Analysis of cranial ultrasound images for newborn

**DOI:** 10.3389/fneur.2022.1090275

**Published:** 2023-01-04

**Authors:** Qing Zhang, Xihui Zhou

**Affiliations:** ^1^First Affiliated Hospital of Xi'an Jiaotong University, Xi'an Jiaotong University Health Science Center, Xi'an Jiaotong University, Xi'an, Shaanxi, China; ^2^Northwest Women's and Children's Hospital, Xi'an Jiaotong University Health Science Center, Xi'an, Shaanxi, China

**Keywords:** cranial, ultrasound images, newborn, filtering, feature extraction

## Abstract

**Introduction:**

Neonatal cranial ultrasound plays an important role in the evaluation of neonatal brain development and related diseases.

**Methods:**

This paper preliminarily explored the analysis and interpretation methods of neonatal brain ultrasound images, and applied the relevant medical image analysis methods to analyze the relevant neonatal brain ultrasound images in more detail.

**Results:**

Compared with other types of imaging methods, ultrasound has its unique advantages and characteristics in such applications as neonatal head imaging.

**Discussion:**

The analysis steps and schemes adopted in this paper have certain reference significance for the analysis of other neonatal brain pictures.

## 1. Introduction

Premature and other types of critically ill newborns are increasing. Neonatal intracranial hemorrhage, hypoxic ischemic encephalopathy, intracranial infection, hydrocephalus, leukomalacia and other brain injury diseases threaten the health of children. Ultrasound has the advantages of non-invasiveness and convenience, and is an important method for analyzing neonatal brain diseases. On the other hand, neonatal intracranial diseases can lead to neurological sequelae, seriously affecting the prognosis of sick newborns. The quantitative analysis of neonatal brain ultrasound has important reference significance for the screening of neonatal diseases, and can be used as an important auxiliary means for neonatal related neurological and developmental diseases.

In Pellicer et al. (1), authors studied the global and regional postnatal cerebral circulatory changes, this research has important reference significance for relevant fields. Doppler imaging of preterm infants is an important means to identify some diseases of preterm infants, which is helpful to evaluate the risk and progress of diseases (2). In Neil and Inder (3), several main methods of infant brain imaging were compared, with particular attention paid to the correlation between imaging results and neural development. In ultrasound examination for neonates, it is usually necessary to divide key areas, including ventricle, brain parenchyma, sulcus and gyrus, so as to make a comprehensive judgment on diseases (4); the work in this paper also attempts to analyze relevant medical image data from multiple perspectives. It is worth mentioning that compared with ultrasound, MRI can also be used to explore the relevant lesions (5). Neonatal cranial ultrasound can be used repeatedly as needed to analyze the time and change process of brain injury; Compared with other neuroimaging methods, cranial ultrasound is relatively cheap and easier to detect some abnormalities (6). The cranial ultrasound examination of neonates is reliable and easy to obtain. The proper setting of the machine can effectively improve the diagnostic value; It should be pointed out that, although neonatal cranial ultrasound has many advantages, it also has some limitations (7). Ultrasound examination of premature infants is also an important means to examine the skull of premature infants. Through the development of a unified program, we hope to find skull ultrasound abnormalities at an early stage (8). Through multiple scans, we can screen the abnormal patterns of head ultrasound in premature infants and analyze the relevant perinatal risk factors (9). Cranial ultrasound plays an important role in the whole research process. Compared with MRI and CT, neonatal brain ultrasound is applied earlier, which helps reduce the demand for other imaging methods (10). On the one hand, cranial ultrasound is an excellent tool to detect brain abnormalities in premature infants; However, it also has its limitations, and some disease types also need MRI assistance (11). In Africa, cranial ultrasound is also an important means of detecting neonatal brain injury (12). In addition to the image method to recognize the symptoms, there are other methods that integrate machine learning algorithms, which have important enlightenment for this paper (13–15). To sum up, despite certain limitations, neonatal cranial ultrasound is still an important means to explore various diseases of newborns. Neonatal intracranial hemorrhage is characterized by hyperechoic reflex. Brain edema is an early pathological change of hypoxic—ischemic encephalopathy. By analyzing the corresponding neonatal ultrasound images, this article explores effective methods and ideas of symptom detection, which has certain reference significance for the screening of neonatal ultrasound diseases.

The main trends of medical imaging are: expansion of software applicability, detection of various diseases, integration with imaging equipment, etc. The main differences between medical images and general images are: (1) different dynamic ranges; (2) Medical images contain many image modes; (3) Noise caused by imaging principle. In recent years, with the development of machine learning and artificial intelligence, brain oriented medical image classification and recognition methods have also shown a rapid development trend. For example, using convolutional neural network, specific diseases can be identified through MRI images of the brain. The 3D convolution neural network can also be used to diagnose cerebral microbleeds, relying on MRI images. For ultrasound images, researchers have also carried out a series of explorations in order to more comprehensively and accurately analyze and identify the symptoms. The main methods include the method based on region growth, the method based on edge detection, the medical ultrasonic image segmentation method based on deformable model, the segmentation technology based on fuzzy set theory, and the segmentation technology based on neural network. In the previous analysis, it was pointed out that ultrasound examination is an important means of neonatal brain disease screening. Based on this conclusion, this paper presented several basic steps of skull ultrasound image analysis, and analyzed the relevant images and results in more detail.

## 2. Basic principles and steps of the method

First, we need to perform simple physical cropping of the image to eliminate interference and retain useful ultrasonic parts. The ultrasonic image after simple shearing contains noise and is not prominent enough in visual details. Therefore, we also perform adaptive median filtering and adaptive histogram equalization with limited contrast on the sheared ultrasonic image.

**Figure 1:**
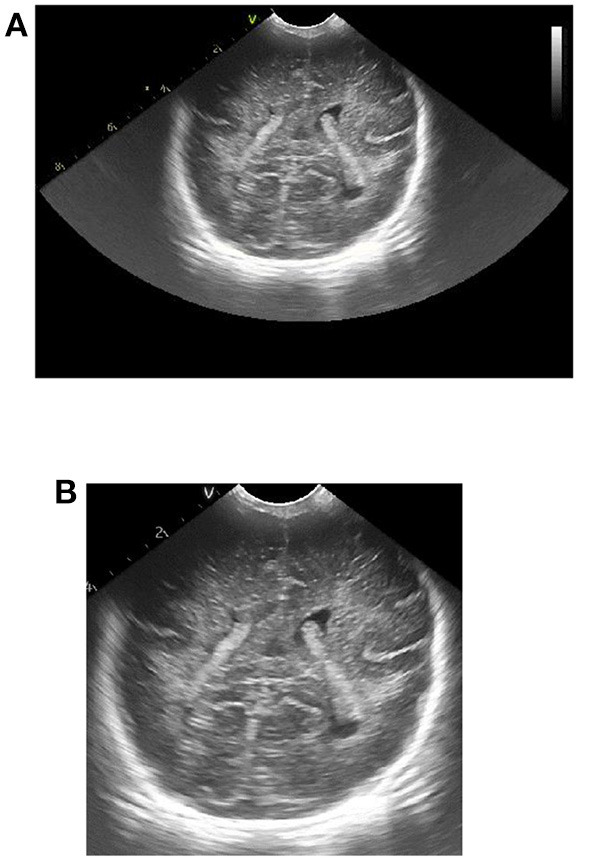
Effect picture of ultrasonic image clipping. **(A)** Ultrasound image before clipping, **(B)** cropped ultrasound image.

To remove additional information around the initial ultrasound image, we physically cut it roughly. The gray value of the image is read row by row and column by column. The number of pixels with gray value of 0 in each row and column is calculated, and the corresponding threshold is set to obtain the coordinate values corresponding to the starting point and the cutoff point of the clipping region. By cutting the original ultrasound image according to the obtained coordinate value, the interference can be removed and useful information can be retained. The image contrast effect before and after clipping is shown in Figure 1.

Due to the inherent characteristics of ultrasound imaging, ultrasound images usually contain some noise and artifacts. The existence of these noises and artifacts not only obscures the characteristics of low-contrast tissues, making it difficult to identify some small structures, but also reduces the ability of observers to analyze detailed features to a certain extent, which has a great impact on the distinction between normal tissues and pathological tissues. In addition, there may be some interfering pseudo contours in the ultrasound image, which will interfere with the subsequent image processing. Therefore, in order to improve the image quality, it is necessary to filter and denoise the original ultrasonic image. However, in the field of medical ultrasound, it is generally believed that the distribution and shape of noise and artifacts in ultrasound images contain relevant information useful for diagnosis. Therefore, the filtering of medical ultrasound images needs to achieve the effect of effectively suppressing noise and retaining some useful micro-tissue information. For the brain ultrasound images of premature infants, we chose the adaptive median filtering method for processing.

Median filtering is a classical non-linear filtering method. This filtering method introduces the idea of classification statistical theory into the calculation, which can effectively filter out isolated noise points. The central idea is to statistically sort the pixels near a certain point in the image according to the gray level, obtain the median value of the pixel gray value in the permutation sequence, and then replace the original pixel value of the point with the median value, so as to effectively filter the points with a large difference in the gray value of the surrounding pixels.

It can be seen from the central idea of median filtering that the size of the selected template window affects the effect of median filtering. If the selected template window is too large, it will cause serious loss of image details; If the selected template window is too small, the noise cannot be filtered effectively. The adaptive median filtering algorithm can automatically match the corresponding filtering window according to the change of noise concentration. Compared with the standard median filter, the adaptive median filter has significantly improved in terms of denoising and image detail retention. The basic principle of the algorithm is as follows: the adaptive median filtering algorithm has two processing layers, Level A and Level B. In Level A, if *z*_min_ < *z*_*med*_ < *z*_max_, it means that the pixel gray value in the selected template window is not noise, at this time, it is necessary to jump to Level B for judgment. If *z*_min_ < *z*_*med*_ < *z*_max_, it is needed to increase the size of the template window. If the size of the template window is less than or equal to the maximum window size, repeat Level A, otherwise output *z*_*med*_. In level B, if *z*_min_ < *f*(*m, n*) < *z*_max_, then the output is *f*(*m, n*), otherwise the output is *z*_*med*_.

**Figure 2:**
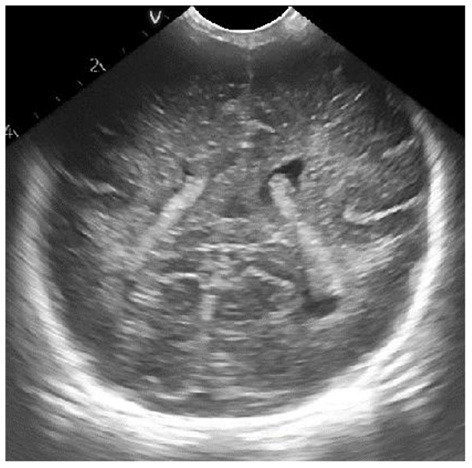
Ultrasonic image after adaptive median filtering.

The ultrasonic image after adaptive median filtering is shown in Figure 2. It can be seen from the figure that the ultrasonic image after the adaptive median filter processing is smoother, most of the noise can be effectively filtered, and the boundary information of the high echo area can be completely preserved.

Ultrasonic images obtained from ultrasonic equipment usually have poor contrast, which makes it difficult to perform subsequent feature extraction and recognition analysis. Therefore, we need to enhance the contrast of the ultrasound image after adaptive median filtering in order to improve the image clarity, improve the visual effect of the image and make the details of the ultrasound image more prominent. For the brain ultrasound image, we choose the limited contrast adaptive histogram equalization method to enhance its contrast.

Gray histogram is the function of gray distribution which is the statistics of gray distribution in image. It represents the correspondence between each gray level and the occurrence frequency of the gray level in the digital image. Generally, the gray value of the image with darker visual effect is mainly concentrated on the left side of the gray histogram, while the gray value of the image with brighter visual effect is mainly concentrated on the right side of the gray histogram. By performing histogram equalization on images with poor visual effects, the grayscale distribution in the image can be adjusted so that the histogram basically covers the entire grayscale value and the distribution is basically uniform, thereby achieving the effect of enhancing image contrast.

The basic idea of histogram equalization is to obtain the corresponding gray value s of the balanced image by some transformation function T of the gray value r of the original input image. Usually, the transformation function T needs to satisfy the following two conditions: first, the transformation function T needs to increase monotonically in the whole range of gray value, and the gray value of the point with larger gray value in the original image is still larger after transformation; second, after the transformation function T, the value range of the original gray value will not change.

For example, for an image of size, because its gray level is a discrete value within the range [0, L − 1], Therefore, it can be histogram equalized.


(1) $$\begin{array}{l} \begin{array}{lll} s_{k}=T(r_{k})=(L-1)\overset{k}{\underset{j=0}{\sum^{\text{​}}}}p_{r}(r_{j}) & = & \frac{L-1}{MN}\overset{k}{\underset{j=0}{\sum^{\text{​}}}}n_{j}, \end{array}\\ \;k=0,1,2,\cdots L-1 \end{array}$$

Where, *MN* is the number of pixels of the whole image, *n*_*k*_ indicates the number of pixels with gray value *r*_*k*_, *L* indicates the number of gray levels in the whole image.

Histogram equalization makes the same histogram transformation for the pixels of the whole image, which is beneficial to the image with balanced pixel value distribution, but it cannot play a good enhancement effect, and is not suitable for craniocerebral ultrasound images with bright areas and dark areas. To this end, we use an adaptive histogram equalization method with limited contrast to improve the local over-enhancement problem of the image, and can also effectively limit the excessive amplification of noise in the image. The specific steps of this method are as follows:

- Divide the original image equally. After segmentation, the size of each sub block is *m* × *n*. The larger the sub block after segmentation, the more obvious the effect of image contrast enhancement, but more details will be lost. The size of the divided sub block is usually set to 8 × 8.
- Calculate the gray level histogram of each sub block, and make the gray level in each sub block obtain the same number of pixels, that is, the average number of pixels $\bar{G}$:

(2) $$\begin{array}{l} \bar{G}=\frac{m\times n}{G_{n}} \end{array}$$

Where, *G*_*n*_ represents the number of gray levels in the n^th^ sub block.

- Calculate the truncation value *C* of each sub block, then successively intercept the number of pixels in the gray histogram of each sub block greater than the truncation value, and calculate the total number of pixels *S* in the intercepted part.
- Reassign the number of truncated pixels to the gray levels of the histogram, and calculate the number of truncated pixels allocated to each pixel level *A*:
  (3) $$\begin{array}{l} A=\frac{S}{G_{n}} \end{array}$$
- Histogram equalization is performed on the new cut gray level histogram obtained from each sub block.
- Each sub block is traversed, and bilinear interpolation between blocks is performed. When histogram equalization is performed after each sub block is truncated, the corresponding relationship between pixels is different, so the enhanced image will have blocking effect. Therefore, it is necessary to take the gray value of the central pixel of each sub block as the reference point, and recalculate the enhanced gray value of other pixel points in the image through the bilinear interpolation method.

The following figure shows the processing effect comparison between adaptive histogram equalization with limited contrast and ordinary histogram equalization. From this map, it can be seen that compared with ordinary histogram equalization, contrast-limited adaptive histogram equalization can not only significantly enhance the darker areas in the image, but also not over-enhance the brighter areas in the image, and effectively enhance the contrast of the entire image, meeting the needs of contrast enhancement of craniocerebral ultrasound images.

In multimedia data such as brain ultrasound images, there is usually a lot of information, and only a small part of this information is useful information we want, and most of the data information is unnecessary or even useless to us. If we process all the data in the brain ultrasound image in the same way, it will undoubtedly increase the complexity of the operation and cause a lot of waste of resources. Therefore, we need to pay different attention to different regions in brain ultrasound images. Typically, we name regions of interest (ROI) as those that contain useful information and need the most attention.

ROI extraction is of great significance in brain ultrasound image analysis. Accurate, effective and rapid extraction of brain ultrasound image ROI for subsequent quantitative analysis of ultrasound images laid a solid foundation. At present, there are two main methods for region of interest extraction: First, based on the visual characteristics of the human eye, the human eye perception mechanism is used to obtain the visual sensitive region, and the region of interest is extracted by manual interaction. The method is user-centered, and the extracted region of interest is relatively accurate, efficient and accurate. The second is to use image segmentation technology to automatically extract the region of interest. According to the special needs of the selected brain ultrasound image, we choose to use manual interaction to extract the region of interest in the image.

The process of extracting ROI based on manual interaction is as follows:

- Read the brain ultrasound image after the adaptive histogram equalization processing with limited contrast;
- Call the “freehand” function box provided in MATLAB to select the part of interest;
- Call the built-in “createMask” function of MATLAB to create a mask for the region of interest selected by the frame. After the creation, set the value of the selected part of interest to 1 and the value of the rest to 0;
- Call the “contour” function of MATLAB to obtain the boundary of the created mask area, and display it in the same picture with the original brain ultrasound image, that is, the extraction of the region of interest is completed. The final extraction result is shown in the following figure.

According to the special requirements for processing the brain ultrasound image, we need to select a reference area besides extracting the region of interest. For the reference area, we choose to use a circle with fixed size for frame selection. The frame selection process is similar to the extraction process of the region of interest. Just change the function called in step (2) to “ellipse”, and set the starting position of the reference area and the required circle size in this function. The reference area selected in the final box is shown in the figure below.

The quantitative analysis of ultrasound image specifically refers to the detection and measurement of the regions of interest in the ultrasound image to obtain their objective information, and then objectively establish the description of the image. Quantitative analysis of ultrasonic images is a process from images to data, which requires necessary statistical knowledge. In this quantitative analysis of brain ultrasound images, we mainly carried out from two aspects: quantitative analysis of echo intensity of white matter and quantitative analysis of echo homogeneity of white matter.

The quantitative analysis of the echo intensity of the white matter of the brain is mainly based on the grayscale characteristics of the ultrasound image. The gray feature takes the gray value of the pixels of the ultrasound image itself as the key feature, which is the most basic feature in the image. It describes the surface features of an object in an image with a grayscale range of 0–255. The commonly used gray feature parameters are: gray mean, gray standard deviation, distortion and entropy. The quantitative analysis of brain ultrasound white matter echo intensity mainly uses the parameters of gray mean. The calculation formula is as follows:


(4) $$\begin{array}{l} M=\overset{L-1}{\underset{r=0}{\sum }}rp\left(r\right) \end{array}$$

Where, *M* refers to the average gray level, *L* refers to the gray level of the image, *r* refers to the pixel value, and *p (r)* refers to the probability that the gray level is *r*.

The specific process of quantitative analysis of echo intensity of white matter in brain is as follows:

- Select the region of interest and the corresponding reference region in the brain ultrasound image after the limited contrast adaptive histogram equalization processing;
- Calculate the average pixel gray value of the region of interest and the reference region;
- Divide the average pixel gray value of the region of interest by the average pixel gray value of the selected reference region to obtain the relative gray value of the echo of the region of interest;
- According to medical common sense, analyze the relative gray value of the echo of the region of interest.

**Figure 3:**
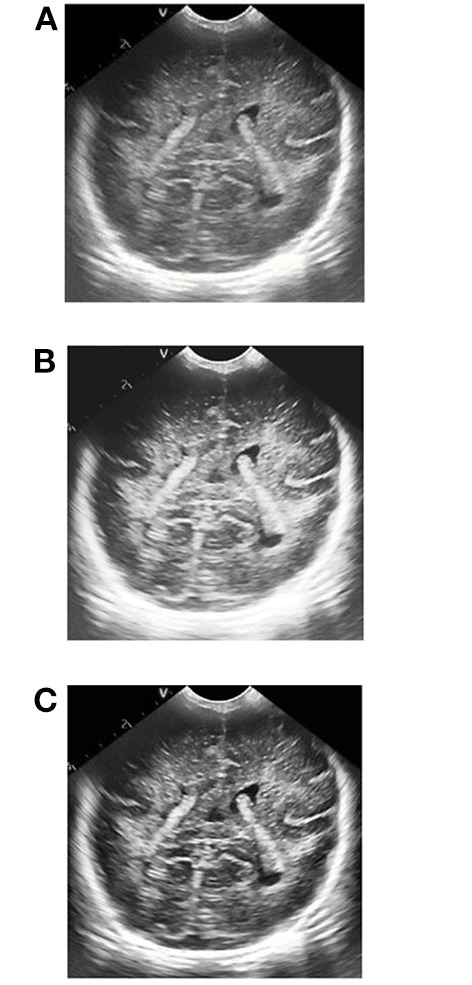
Contrast enhancement effect pictures of different methods. **(A)** Original picture, **(B)** effect picture of histogram equalization, **(C)** CLAHE effect picture.

**Figure 4:**
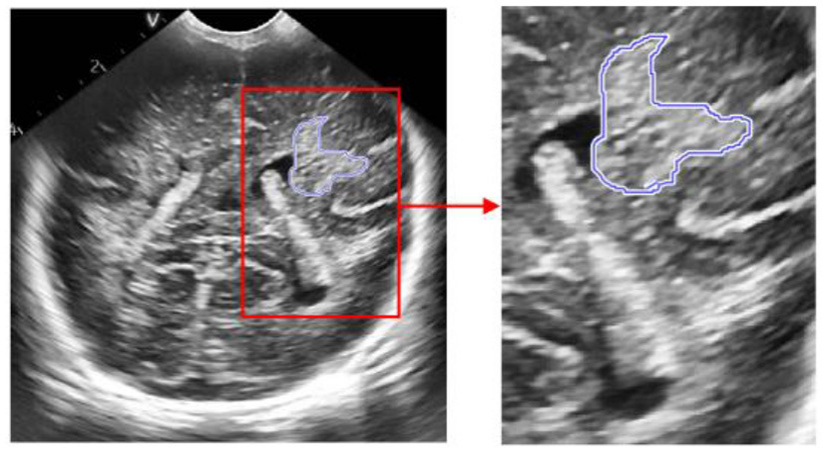
Schematic diagram of region of interest frame selection process.

**Figure 5:**
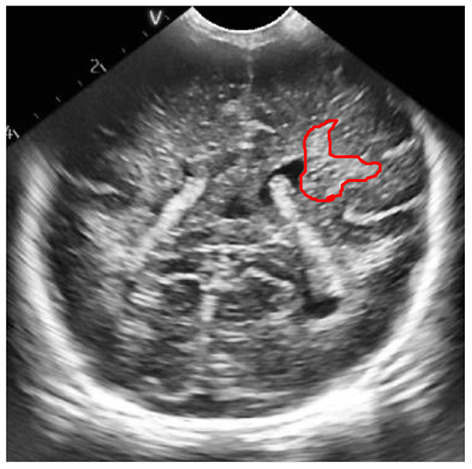
ROI extraction result map.

**Figure 6:**
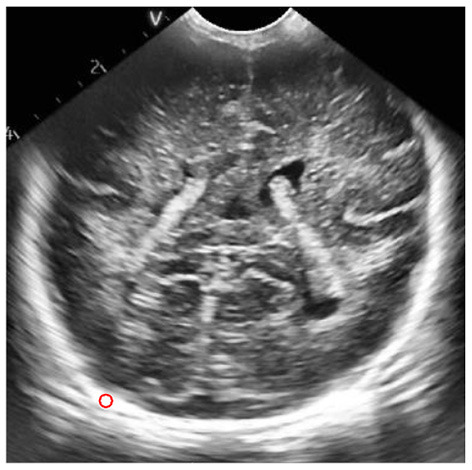
Results of frame selection of reference area.

**Figure 7:**
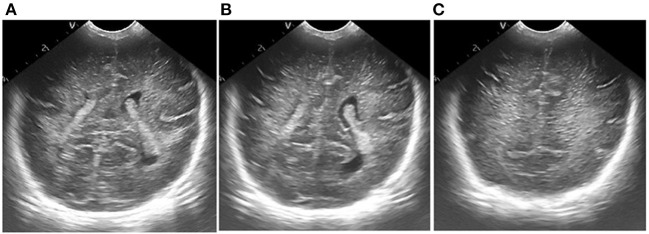
Results of ultrasonic image clipping. **(A)** Plane 1, **(B)** plane 2, **(C)** plane 3.

**Figure 8:**
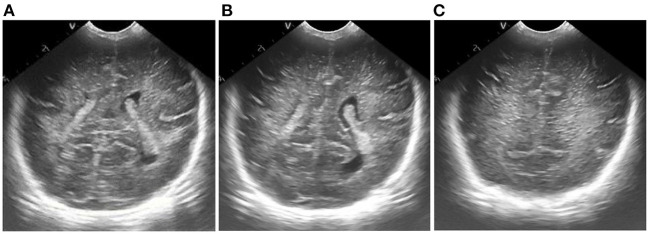
Results of adaptive median filtering. **(A)** Plane 1, **(B)** plane 2, **(C)** plane 3.

**Figure 9:**
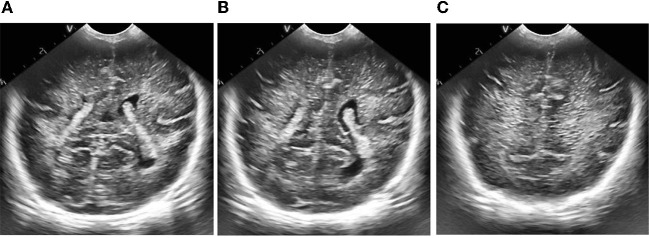
Results of adaptive histogram equalization with limited contrast. **(A)** Plane 1, **(B)** plane 2, **(C)** plane 3.

**Figure 10:**
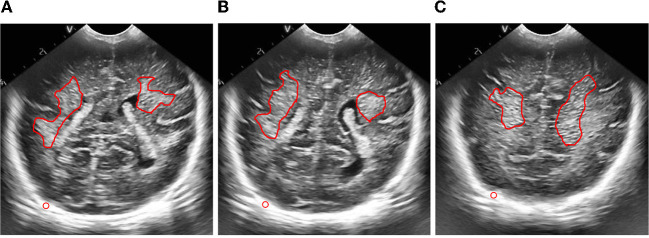
Figure of result of frame selection of regions of interest and reference regions. **(A)** Plane 1, **(B)** plane 2, **(C)** plane 3.

The quantitative analysis of echo homogeneity of white matter is based on the texture characteristics of ultrasound images. Texture is a visual feature that reflects the homogeneous phenomenon in an image. It reflects the structural organization and arrangement attributes of the object surface with slow or periodic changes. Texture features usually have the following three signs: ① some local sequence is repeated constantly; ② Non-random arrangement; ③ Roughly uniform unity in the texture area. Different from gray scale, color and other image features, texture features are mainly represented by the gray distribution of pixels and their surrounding space fields. In image analysis, common texture features include gray level co-occurrence matrix, gray level run matrix and gray level difference matrix, among which the effect of image texture analysis based on gray level co-occurrence matrix is better. Therefore, we choose the texture feature of gray level co-occurrence matrix to quantitatively analyze the degree of echo homogeneity of white matter. The image processing steps and processes involved in the paper are shown in Figures 3–10.

## 3. Partial results and discussion

According to the selected scheme, we processed the brain ultrasound images of the same subject in three different planes, and the whole processing process was completed based on MATLAB software. According to the results, we also carried out the quantitative analysis of echo intensity and echo homogeneity of white matter.

First of all, we preprocessed three different planes of brain ultrasound images of the same subject, and the results are as follows:

Then, we extracted the region of interest and selected the reference region of the ultrasonic image after preprocessing.

According to the data requirements of quantitative analysis of echo intensity of brain white matter and quantitative analysis of echo homogeneity of brain white matter, we calculated the average pixel gray value of regions of interest and reference regions, the relative gray value of regions of interest, and the statistical characteristics of gray level co-occurrence matrix generated in different directions in these three planes. The calculation results are as follows:

## 4. Conclusion

In order to quantitatively analyze the echo intensity of white matter in brain ultrasound images, we can see from the data in Tables 1–3 that in the regions of interest depicted in different three planes, the relative gray value of the subjects is higher, that is, the echo intensity of the corresponding white matter region is significantly enhanced. In order to quantitatively analyze the homogeneity of white matter echo in brain ultrasound images, from the statistical characteristics of the gray level co-occurrence matrix generated in different directions in Tables 4–6, it can be seen that the homogeneity of the region of interest in the three ultrasound images is poor, that is, the white matter in the corresponding white matter region of the subject is heterogeneous. Based on medical knowledge associated with brain ultrasound images, it can be determined that the subject has a brain injury disease.

**Table 1:** Relevant calculation data for quantitative analysis of echo intensity of white matter in plane one brain.

**Average pixel gray value of ROI 1:**	**144.2773**
Average pixel gray value of ROI 2:	153.8880
Average pixel gray value of reference area:	252.7574
Relative gray value of ROI 1:	57.0813
Relative gray value of ROI 2:	60.8837

**Table 2:** Relevant calculation data for quantitative analysis of echo intensity of white matter in plane two.

**Average pixel gray value of ROI 1:**	**146.3016**
Average pixel gray value of ROI 2:	159.7064
Average pixel gray value of reference area:	252.9822
Relative gray value of ROI 1:	57.8308
Relative gray value of ROI 2:	63.1295

**Table 3:** Relevant calculation data for quantitative analysis of echo intensity of white matter in plane three brain.

**Average pixel gray value of ROI 1:**	**160.5301**
Average pixel gray value of ROI 2:	143.6090
Average pixel gray value of reference area:	253.5148
Relative gray value of ROI 1:	63.3218
Relative gray value of ROI 2:	56.6472

**Table 4:** Relevant calculation data for quantitative analysis of echo homogeneity of white matter in plane one brain.

	**E**	**N–E**	**N**	**N–W**	**W**	**S–W**	**S**	**S–E**	**E–W**	**N–S**	**Cross**	**Circle**
ENE1	0.1445	0.0936	0.1152	0.1329	0.1445	0.0936	0.1152	0.1329	0.1445	0.1152	0.1299	0.1216
IDM1	0.8645	0.7328	0.8027	0.8438	0.8645	0.7328	0.8027	0.8438	0.8645	0.8027	0.8336	0.8110
CON1	0.2815	0.7587	0.4661	0.3251	0.2815	0.7587	0.4661	0.3251	0.2815	0.4661	0.3738	0.4579
ENT1	3.2778	3.8747	3.5914	3.3678	3.2778	3.8747	3.5914	3.3678	3.2778	3.5914	3.4346	3.5279
ENE2	0.1295	0.1142	0.1039	0.0829	0.1295	0.1142	0.1039	0.0829	0.1295	0.1039	0.1167	0.1076
IDM2	0.8504	0.8135	0.7860	0.7105	0.8504	0.8135	0.7860	0.7105	0.8504	0.7860	0.8182	0.7901
CON2	0.3128	0.4116	0.5246	0.8519	0.3128	0.4116	0.5246	0.8519	0.3128	0.5246	0.4187	0.5252
ENT2	3.3915	3.5550	3.7006	3.9894	3.3915	3.5550	3.7006	3.9894	3.3915	3.7006	3.5460	3.6591

**Table 5:** Relevant calculation data for quantitative analysis of echo homogeneity of white matter in plane two.

	**E**	**N–E**	**N**	**N–W**	**W**	**S–W**	**S**	**S–E**	**E–W**	**N–S**	**Cross**	**Circle**
ENE1	0.1423	0.0884	0.1107	0.1340	0.1423	0.0884	0.1107	0.1340	0.1423	0.1107	0.1265	0.1189
IDM1	0.8668	0.7232	0.7992	0.8524	0.8668	0.7232	0.7992	0.8524	0.8668	0.7992	0.8330	0.8104
CON1	0.2772	0.8226	0.4821	0.3055	0.2772	0.8226	0.4821	0.3055	0.2772	0.4821	0.3796	0.4719
ENT1	3.3212	3.9847	3.6685	3.3817	3.3212	3.9847	3.6685	3.3817	3.3212	3.6685	3.4949	3.5890
ENE2	0.1412	0.1218	0.1108	0.0923	0.1412	0.1218	0.1108	0.0923	0.1412	0.1108	0.1260	0.1165
IDM2	0.8611	0.8202	0.7933	0.7327	0.8611	0.8202	0.7933	0.7327	0.8611	0.7933	0.8272	0.8018
CON2	0.2826	0.4066	0.4931	0.7288	0.2826	0.4066	0.4931	0.7288	0.2826	0.4931	0.3879	0.4778
ENT2	3.2760	3.4962	3.6250	3.8594	3.2760	3.4962	3.6250	3.8594	3.2760	3.6250	3.4505	3.5642

**Table 6:** Relevant calculation data for quantitative analysis of echo homogeneity of white matter in plane three brain.

	**E**	**N–E**	**N**	**N–W**	**W**	**S–W**	**S**	**S–E**	**E–W**	**N–S**	**Cross**	**Circle**
ENE1	0.1164	0.0672	0.0810	0.0901	0.1164	0.0672	0.0810	0.0901	0.1164	0.0810	0.0987	0.0887
IDM1	0.8475	0.6754	0.7416	0.7761	0.8475	0.6754	0.7416	0.7761	0.8475	0.7416	0.7946	0.7602
CON1	0.3192	1.1057	0.7312	0.5468	0.3192	1.1057	0.7312	0.5468	0.3192	0.7312	0.5252	0.6757
ENT1	3.5205	4.2736	4.0292	3.8551	3.5205	4.2736	4.0292	3.8551	3.5205	4.0292	3.7749	3.9196
ENE2	0.1125	0.0825	0.0750	0.0627	0.1125	0.0825	0.0750	0.0627	0.1125	0.0750	0.0937	0.0832
IDM2	0.8525	0.7612	0.7307	0.6685	0.8525	0.7612	0.7307	0.6685	0.8525	0.7307	0.7916	0.7532
CON2	0.3083	0.6506	0.8302	1.1954	0.3083	0.6506	0.8302	1.1954	0.3083	0.8302	0.5692	0.7461
ENT2	3.5875	4.0420	4.1858	4.4070	3.5875	4.0420	4.1858	4.4070	3.5875	4.1858	3.8866	4.0556

## Data availability statement

The raw data supporting the conclusions of this article will be made available by the authors, without undue reservation.

## Author contributions

QZ: image analysis. XZ: research guidance. All authors contributed to the article and approved the submitted version.
